# Nerve Stretching for the Relief of Neuralgia

**Published:** 1878-05

**Authors:** 


### NERVE STRETCHING FOR THE RELIEF OF NEURALGIA.

In the *British Medical Journal* for December two cases of nerve stretching are reported:

The first was under Mr. Heath's care. The patient, a stout woman, had long suffered intense neuralgic pains in the right buttock, and down the back of right thigh. Nothing relieved the sciatica, though all the usual remedies were diligently tried. Mr. Heath cut down upon the sciatic nerve, and exposed it where it emerges from beneath the gluteus maximus. Seizing it with two forceps, he forcibly stretched it, and loosened it from its bed. No inconvenience followed the operation, and the woman has since been perfectly free from pain; in fact, it promises to be a complete success. The second case was that of a man under Mr. Bradley's care, suffering from hyperkinesis of the spinal system generally. The condition of the poor fellow was so distressing that Dr. Ross, who had had previous charge of the case, suggested that nerve stretching should be systematically tried, first in the legs, and then in the arms. As the operation was quite of a tentative character, the only nerve stretched at the first operation was the anterior crural, just beyond Poupart's ligament. It is too recent to speak confidently about the result, but so far as it has gone, it is encouraging. There is no loss of power or sensation in the muscles supplied by the anterior crural, while there is a marked diminution in the choreic movements of the parts.

